# IL-11 Is Elevated and Drives the Profibrotic Phenotype Transition of Orbital Fibroblasts in Thyroid-Associated Ophthalmopathy

**DOI:** 10.3389/fendo.2022.846106

**Published:** 2022-02-22

**Authors:** Pengsen Wu, Bingying Lin, Siyu Huang, Jie Meng, Fan Zhang, Min Zhou, Xiangqing Hei, Yu Ke, Huasheng Yang, Danping Huang

**Affiliations:** State Key Laboratory of Ophthalmology, Zhongshan Ophthalmic Center, Sun Yat-sen University, Guangdong Provincial Key Laboratory of Ophthalmology and Visual Science, Guangzhou, China

**Keywords:** clinical activity score, fibrosis, IL-11, orbital fibroblast, thyroid-associated ophthalmopathy

## Abstract

Orbital fibrosis is a hallmark of tissue remodeling in thyroid-associated ophthalmopathy (TAO). Previous studies have shown that interleukin (IL)-11 plays a pivotal profibrotic role in various inflammatory and autoimmune diseases. However, the expression pattern of IL-11 in patients with TAO and whether IL-11 is mechanistically linked with pathological fibrosis remains unknown. In this study, we investigated IL-11 levels in the serum and orbital connective tissue of patients with TAO, and evaluated the correlation of these levels with the patient’s clinical activity score. We also evaluated the expression pattern of IL-11Rα in orbital connective tissue. Furthermore, we elucidated the regulatory factors, profibrotic function, and downstream signaling pathways for IL-11 in TAO using *in vitro* studies. IL-11 levels in serum and orbital connective tissues were increased in patients with TAO, as compared with healthy controls. In addition, both levels were positively correlated with disease activity. Single-cell RNA sequencing of orbital connective tissue indicated that IL-11Rα was dominantly expressed in orbital fibroblasts (OFs). RNA sequencing of paired unstimulated and transforming growth factor (TGF)-β1-stimulated samples demonstrated that upregulation of IL-11 expression defined the dominant transcriptional response. IL-11 signaling was also confirmed to be downstream of TGF-β1 and IL-1β. Therefore, we deduced that IL-11 protein is secreted in an autocrine loop in TAO. We also indicated that IL-11 mediated the profibrotic phenotype switch by inducing the expression of myofibroblast differentiation markers, including α-smooth muscle actin and collagen type I α1, which could be abrogated by an anti-IL-11 neutralizing antibody. Furthermore, we revealed that extracellular regulated protein kinase may be a crucial factor in the pro-fibrotic, translationally specific signaling activity of IL-11. These data demonstrate that IL-11 plays a crucial role in orbital fibroblast phenotype switching and may be a potential therapeutic target candidate for the treatment of TAO.

## Introduction

Thyroid-associated ophthalmopathy (TAO) is an organ-specific autoimmune ocular disease related to dysregulated thyroid gland activity, particularly Graves’ hyperthyroidism, and is thus also called Graves’ orbitopathy. As the major extrathyroidal manifestation of Graves’ disease (GD), TAO remains an intractable disorder most frequently occurring in patients with GD (1, 2). The main pathological process involves inflammatory infiltration of orbital tissue, extracellular matrix (ECM) production, orbital tissue remodeling, and orbital volume expansion, which provide a mechanical foundation for illustrating the clinical features of periorbital edema, conjunctival hyperemia, eyelid retraction, diplopia, protrusion, exposure keratitis, and compressive optic neuropathy (1, 3–5). This appearance-destroying and vision-threatening ocular disease seriously affects the quality of patients’ lives.

Orbital fibrosis is a hallmark of pathological changes in TAO (6). Orbital fibroblasts (OFs), which act as both target cells and the main effector cells, are essential mediators of fibrotic processes (7, 8). By interacting with T cells and autoantibodies produced by B cells in a complex biological network, resident OFs are subsequently activated, promoting the release of copious inflammatory mediators, overproduction of ECM, and phenotypic transition into myofibroblasts (9). The pathological hallmarks of orbital fibrosis in TAO are centered on myofibroblasts (10), which display two typical features: secretion of ECM and contractility, with the expression of α-smooth muscle actin (α-SMA), resulting in excess accumulation of ECM proteins and increased tissue stiffness (11, 12). However, the initial triggers that activate the fibroblast-to-myofibroblast transition in TAO remain unclear.

Interleukin-11 (IL-11) belongs to the interleukin-6 (IL-6) family of cytokines that share a common β-receptor subunit, glycoprotein 130 (gp130) (13). IL-11 can be secreted by various cell types including fibroblasts, epithelial cells, osteoblasts, endothelial cells, neurons, endometrial cells and lung smooth muscle cells (14). By binding to its specific receptor alpha subunit (IL11Rα) and cognate gp130 receptor, IL-11 can activate downstream canonical (JAK/STAT) and non-canonical (e.g., ERK) signaling pathways. In contrast, the specific IL11Rα receptor is predominantly expressed on fibroblasts (15). Consequently, primary cells, such as OFs are ideal experimental systems for studying IL-11 signaling *in vitro*.

IL-11 was initially identified as a cytokine that can induce the formation and maturation of megakaryocytes (16–18). Therefore, recombinant human IL-11 (rhIL-11) has been approved for treating chemotherapy-induced thrombocytopenia (19). Further studies indicate that IL-11 participates in tumorigenesis by regulating cell proliferation and migration (13, 20). It was later shown that IL-11 exerts a protective and antifibrotic role in the heart and kidney. Animal studies verified this conclusion by applying IL-11 in experimental models of myocardial ischemia or of glomerulonephritis. These results demonstrated that rhIL-11 protects against ischemia-reperfusion injury and suppresses the production and deposition of ECM (21, 22). In contrast, recent studies have revealed that IL-11 plays a pivotal role in the pathological development of cardiovascular fibrosis by inducing excess expression of IL-11 in myofibroblasts in an autocrine signaling loop (23, 24). In addition, a growing body of evidence has confirmed that IL-11 is an essential mediator in a variety of inflammatory or autoimmune diseases including idiopathic pulmonary fibrosis (25), renovascular hypertension (26), nonalcoholic steatohepatitis (27), and systemic sclerosis (28). In TAO, fibrosis accounts for a majority of pathological hallmarks. As IL-11 is a major cytokine that underlies the pathogenesis of fibroproliferative diseases, we hypothesized that IL-11 may also have profibrotic effects in TAO.

In this study, we investigated IL-11 levels in the serum and orbital connective tissues of patients with TAO, and evaluated the correlation of these levels with the clinical activity score (CAS). We also evaluated the expression pattern of IL-11Rα in orbital connective tissue. Furthermore, we elucidated the regulatory factors, profibrotic function, and downstream signaling pathways for IL-11 in TAO using *in vitro* studies.

## Materials and Methods

### Study Participants and Sample Collection

Peripheral blood samples were obtained from TAO patients (n = 40) and healthy volunteers (n = 18), who were recruited from the Zhongshan Ophthalmic Center, Sun-Yat Sen University. Clinical and demographic descriptions of the participating subjects are provided in **Table 1**.

**Table 1:** Demographic Data of Patients with TAO and Controls.

	Patients with TAO	Control subjects	P
No. of participants	40	18	
Sex, M/F	24/16	6/12	0.06
Age, mean ± SD (range), y	50.53 ± 12.57 (24-79)	46.39 ± 20.82 (24-75)	0.602
Clinical activity score			NA
Stable	19		
Active	21		
Duration of TAO, median (range), m	22 (2 - 120)		NA
Therapy history			NA
Thyroid surgery	3/40		
Orbital irradiation	1/40		
Steroid treatment	40/40		
Thyroid function			NA
Hyperthyroid	0/40		
Hypothyroid	3/40		
Euthyroid	37/40		

TAO, Thyroid-associated ophthalmopathy; M, male; F, female; SD, standard deviation; y, year; m, month; NA, not applicable.

Orbital adipose/connective tissues were taken from TAO patients (n=19) during orbital decompression surgery. Control orbital connective tissues were collected from healthy controls (n=10) undergoing blepharoplasty. The baseline characteristics of the TAO patients and healthy subjects are summarized in **Table 2**.

**Table 2:** Demographic Data of Patients with TAO and Controls undergoing surgery.

	Patients with TAO	Control subjects	P
Number of participants	19	9	
Sex, M/F	11/8	4/5	0.505
Age, mean ± SD (range), y	51.11 ± 10.98 (24-65)	49.22 ± 14.03 (35-69)	0.62
Clinical activity score			NA
Stable	13		
Active	6		
Duration of TAO, median (range), m	24 (2-108)		NA
Therapy history			NA
Thyroid surgery	1/19		
Orbital irradiation	1/19		
Steroid treatment	1/19		
Thyroid function			NA
Hyperthyroid	0/19		
Hypothyroid	2/19		
Euthyroid	17/19		

TAO, Thyroid-associated ophthalmopathy; M, male; F, female; SD, standard deviation; y, year; m, month; NA, not applicable.

The enrolled patients had not received any corticosteroid medication, immunosuppressive agents, or radiotherapy for at least 3 months. The exclusion criteria included active infection, recent trauma, a history of other autoimmune diseases, or chronic inflammation. The clinical activity of TAO was graded according to the 7-item CAS scheme proposed by EUGOGO respectively (2).

### Ethical Considerations

Informed consent was obtained from each participant. This study was approved by the Ethics Committee of Zhongshan Ophthalmic Center, Sun-Yat Sen University, and was conducted in accordance with the tenets of the Declaration of Helsinki.

### Cell Culture and Treatment

Orbital adipose/connective tissue was minced into 1–2 mm^3^ pieces, and placed in 10–cm culture dishes containing high glucose Dulbecco’s modified Eagle’s medium (DMEM) supplemented with 20% fetal bovine serum and 1% penicillin/streptomycin (all from Gibco Laboratories, New York, USA), and incubated at 37°C with 5% CO_2._ Culture medium was renewed every 3–4 days. After the cells migrated out of the tissue pieces, OFs were passaged with standard trypsinization techniques when reaching 90% confluence. After passaging, the cells were grown in proliferation medium (DMEM supplemented with 10% FBS and 1% penicillin/streptomycin) using standard cell culture protocols. The cells between passage 3–7 were used for all experiments. The culture medium was replaced with basal medium (DMEM supplemented with 1% FBS and 1% penicillin/streptomycin) 12 hours prior to stimulation. Cells were stimulated with different treatment conditions and durations. Detailed experimental conditions are outlined in the main text or figure legends.

### IL-11 Quantification by ELISA

The IL-11 serum concentrations and concentrations of IL-11 secreted from OFs in the culture supernatants were measured with an enzyme-linked immunosorbent assay (ELISA) kit (RayBiotech, Atlanta, USA) following the manufacturer’s instructions.

### Immunohistochemistry

Paraffin-embedded samples of orbital connective tissues were cut into 3 μm sections and then deparaffinized with xylene, followed by rehydration with graded alcohols. Antigen retrieval was performed by applying citric acid antigen retrieval solution (pH 6.0) to the sections. Endogenous peroxidase activity was blocked using 3% H2O2, and a 3% bovine serum albumin (BSA) block was applied. The sections were treated with primary antibody against IL-11 (Bioss, Beijing, China) overnight at 4°C. Secondary antibodies (Servicebio, Wuhan, China) incubation was performed for 50 minutes at room temperature and then the sections were developed with diaminobenzidine (DAB, Servicebio, Wuhan, China). Subsequently, the cell nuclei were counterstained with hematoxylin, followed by dehydration and sealing with neutral gum. Semi-quantitative analysis of IL-11 density was performed with ImageJ software (National Institutes of Health, Bethesda, USA).

### RNA Sequencing

The total RNA of primary orbital fibroblasts was extracted using an RNA Purification Kit (Yishan Biotechnology, Shanghai, China). RNA extracts were analyzed using an RNA Nano 6000 Assay Kit (Agilent Technologies, CA, USA) in combination with the Bioanalyzer 2100 system. RNA sample preparations were carried out with a total amount of 1 μg RNA per sample. NEBNext^®^ UltraTM RNA Library Prep Kit (Illumina, CA, USA) was used for library preparation and index codes were added to each sample as attribute specifier sequence. After sample clustering, the library preparations were sequenced on an Illumina Novaseq platform. Quality control raw reads were processed using in-house Perl scripts. The reference genome index was built using Hisat2 (v2.0.5) and the paired-end reads were aligned against the reference genome using Hisat2 (v2.0.5) as well. The mapped reads of each sample were assembled by *StringTie* (v1.3.3b) (29). Differential expression testing was performed using the DESeq2 R package (1.20.0). Genes with a P-value < 0.05 as identified by DESeq2 were considered differentially expressed.

### Single Cell RNA Sequencing

Single-cell suspensions from orbital connective tissues were derived from 1 TAO patient and 1 healthy control as previously described (30). Single cells were isolated and lysed with collagenase buffer composed of 5 mg/mL collagenase IV and 10 U/mL DNase I at 37°C for 50 minutes (all from Sigma-Aldrich, St. Louis, USA). Subsequently, filtration and red blood cell removal were performed using the MACS Dead Cell Removal Kit (Miltenyi Biotec, Bergisch Gladbach, Germany). RNA was then reverse-transcribed into cDNA libraries using a 10x Genomics Chromium machine following the manufacturer’s instructions. DNA sequencing was performed on a NovaSeq 6000 Sequencing System (Illumina, CA, USA) using a paired-end 150 sequencing mode. The Illumina output was processed using Cell Ranger 3.0.2 (10×Genomics, CA, USA). Cells of sufficient complexity were clustered and t-distributed stochastic neighbor embedding (t-SNE) plots were generated for visualization using the Seurat R package (31).

### RNA Extraction and Quantitative Real-Time Polymerase Chain Reaction

Following the manufacturer’s instructions, the total RNA was extracted from cultured cells using the RNA Purification Kit, and reverse transcription was performed using an Evo M-MLV RT kit (Accurate Biology, Hunan, China). Quantitative real-time polymerase chain reaction (qRT-PCR) was carried out using SYBR Premix Pro Taq HS (Accurate Biology, Hunan, China) on a Light Cycler 480 Real-Time System (Roche, Basel, Switzerland). GAPDH was selected as the internal control and relative fold change was calculated using the 2^−ΔΔCT^ method. The primers used in this experiment are listed in **Table 3**.

**Table 3:** Primer Sequences of qRT-PCR.

Genes	Sequences (5’ – 3’)
IL-11	F: GTG GCC AGA TAC AGC TGT C
	R: GAA TTT GTC CCT CAG CTG TG
ACTA2	F: GAA CCC TAA GGC CAA CCG GGA GAA A
	R: CCA CAT ACA TGG CGG GGA CAT TGA
COL1A1	F: AAA GAT GGA CTC AAC GGT CTC
	R: CAT CGT GAG CCT TCT CTT GAG
COL1A2	F: CTC CAT GGT GAG TTT GGT CTC
	R: CTT CCA ATA GGA CCA GTA GGA C
FN1	F: CGG TGG CTG TCAGTC AAA G
	R: AAA CCT CGG CTT CCT CCA TAA
GAPDH	F: TTG CCA TCA ATG ACC CCT T
	R: CGC CCC ACT TGA TTT TGG A

F, forward; R, reverse.

### Immunofluorescence

Fibroblasts were seeded on 20**-**mm microscopy culture dishes at approximately 1×10^4^ per well. Cells were fixed at room temperature for 20 minutes using 4% paraformaldehyde, then simultaneously blocked and permeabilized with a solution of 0.3% Triton X-100, 3% BSA, and 5% normal donkey serum (NDS) in phosphate-buffered saline (PBS) for 1 hour at room temperature. Cells were incubated with primary antibodies against IL-11 Rα (R&D Systems, Minneapolis, USA), α-SMA (Abcam, Waltham, USA), collagen type I α1 (COL1A1, Cell Signaling Technology, Beverly, USA) overnight at 4°C, and probed with the Alexa Fluor 488 or Alexa Fluor 546-labeled secondary antibody (both from Nanoprobes, New York, USA) in the dark at room temperature for 1 hour. Nuclei were visualized by staining with DAPI (Bioss, Beijing, China) for 10 min. Finally, an anti-fluorescence quenching agent (Solarbio, Beijing, China) was added, and samples were sealed and mounted. Samples were observed and imaged using a confocal microscope (Carl Zeiss 880, Oberkochen, Germany).

### Immunoblotting

Cell lysates were prepared using RIPA Lysis buffer containing protease and phosphatase inhibitors (all from EpiZyme, Shanghai, China). Protein concentrations were determined using a Bicinchoninic acid (BCA) protein assay kit (Beyotime Biotechnology, Shanghai, China). Protein lysates were separated on 12% polyacrylamide gels and electrotransferred to polyvinylidene fluoride (PVDF) membranes (Millipore, Darmstadt, Germany). Membranes were blocked with QuickBlock™ Blocking Buffer (Genscript, Nanjing, China) for 20 minute and then incubated overnight with primary antibodies against α-SMA, COL1A1, ERK-, Phospho-ERK (p-ERK), STAT3, and phospho-STAT3 (p-STAT3). Glyceraldehyde-3-phosphate dehydrogenase (GAPDH) was used as loading control. All antibodies except α-SMA were from Cell Signaling Technology, Beverly, USA. Subsequently, the membranes were probed with horseradish peroxidase-conjugated mouse or rabbit lg secondary antibodies (both from Cell Signaling Technology, Beverly, USA). Immunoblots were visualized with an enhanced chemiluminescence kit (Millipore, Darmstadt, Germany), and imaged using a chemiluminescence imager (Tanon Science & Technology Co., Ltd., Shanghai, China). Images were subjected to densitometric analysis using ImageJ software.

### Statistical Analysis

Data were analyzed using GraphPad Prism (Prism 8, GraphPad Software, CA, USA). Descriptive statistics were used for the presentation of patient characteristics. Normality was assessed using the Shapiro-Wilk test. Continuous variables with normal distribution are presented as mean ± standard deviation (SD). Discrepancies between the two groups were evaluated using a two-tailed Student’s t-test or Mann-Whitney test when non-normally distributed. A chi-squared test was used to compare categoric variables. One-way analysis of variance (ANOVA), followed by Tukey’s multiple comparison test, was performed for comparisons among multiple groups. Correlation between the two groups was examined using Pearson or Spearman correlation analysis, depending on the data type and distribution. Statistical significance was defined as P values < 0.05.

## Results

### Serum IL-11 Levels Are Elevated in TAO Patients and Correlated With Disease Activity

**Figure 1:**
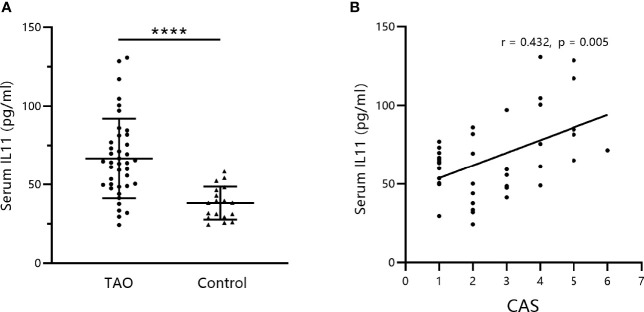
Serum IL-11 levels are elevated in thyroid-associated ophthalmopathy (TAO) patients and correlated with disease activity. **(A)** IL-11 levels were analyzed by enzyme-linked immunosorbent assay in serum obtained from the venous blood of TAO patients and healthy subjects (TAO, n = 40; control, n = 18). The data are expressed as the mean ± standard deviation (SD). ****P < 0.0001. assessed by two-tailed student’s t-test. **(B)** Correlation between IL-11 levels in patients with TAO and the Clinical Activity Score (CAS). Spearman’s test was used for the correlation analysis. P < 0.05 was considered significant.

Initially, circulating IL-11 levels in the serum of patients with TAO and healthy controls were measured by ELISA. We found that serum IL-11 concentrations in patients with TAO were significantly elevated compared to those in healthy controls (**Figure 1A**, mean TAO: 66.6 ± 25.42 pg/mL; mean controls: 38.14 ± 10.49 pg/mL; P < 0.0001). Furthermore, correlation analysis indicated that serum IL-11 levels were positively associated with CAS (**Figure 1B**).

### IL-11 Expression in Orbital Connective Tissues of TAO Patients Is Augmented

**Figure 2:**
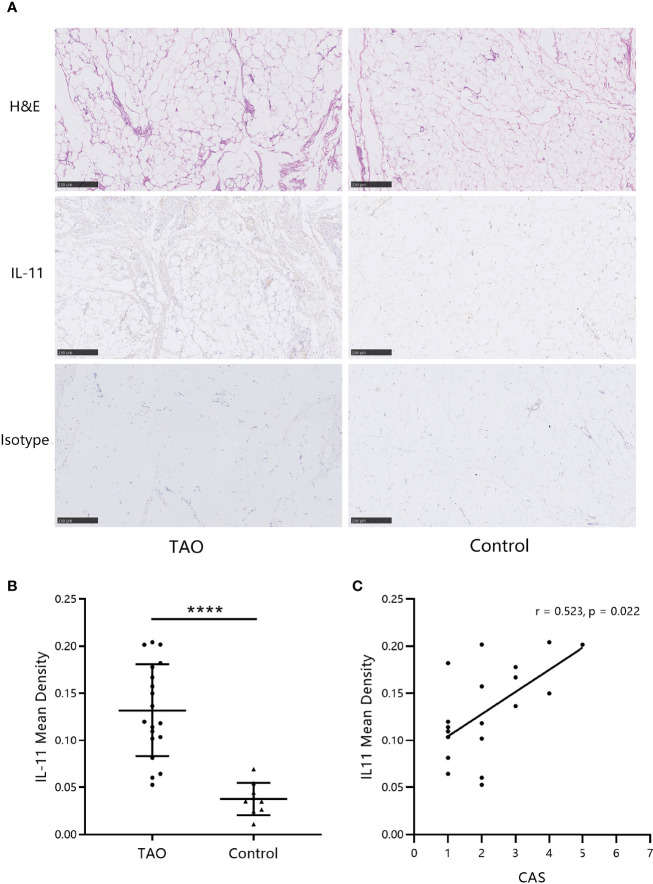
IL-11 expression in orbital connective tissues of thyroid-associated ophthalmopathy (TAO) patients is augmented. **(A)** Immunohistochemistry (IHC) staining was performed on paraffin-embedded biopsy sections. The positive IHC staining of IL-11 was shown as yellowish-brown. One representative staining for each group of biopsies is shown. Scale bars, 250 μm. **(B)** The mean density was used for the semi-quantitative analysis of IHC (TAO, n = 19; control, n = 10). The data are expressed as the mean ± standard deviation (SD). ****P < 0.0001. assessed by two-tailed student’s t-test. **(C)** Correlation between the Clinical Activity Score (CAS) and IL-11 mean density in patients with TAO. H&E, hematoxylin and eosin. Spearman’s test was used for the correlation analysis. P < 0.05 was considered significant.

Next, to assess the levels of IL-11 in orbital connective tissues, immunohistochemistry images were analyzed. As shown in **Figure 2A**, IL-11 expression was augmented in patients with TAO. Statistical analysis revealed significant differences between IL-11 expression in TAO and control subjects (**Figure 2B**). In agreement with systemic IL-11 expression, a positive correlation was detected between IL-11 protein levels in orbital connective tissues and CAS in patients with TAO (**Figure 2C**).

### IL-11 Rα and IL-11 Are Co-Expressed in OFs

**Figure 3:**
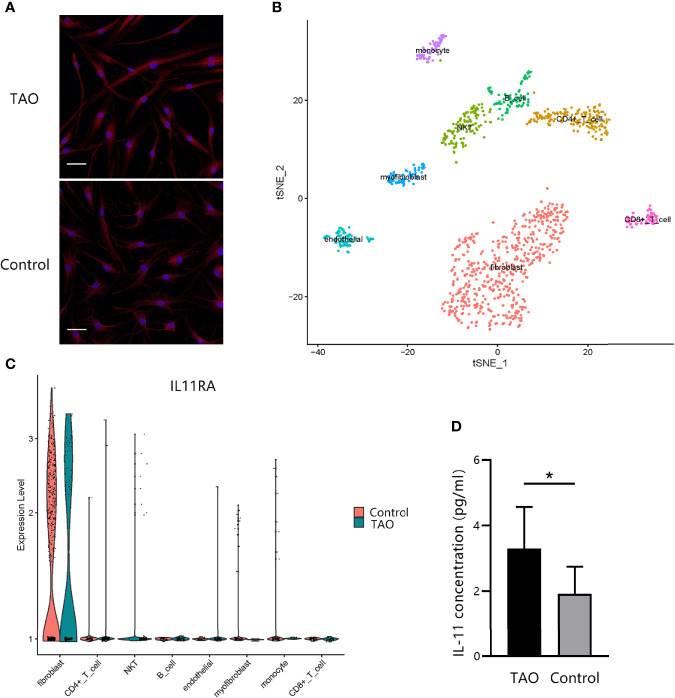
IL-11Rα and IL-11 are co-expressed in orbital fibroblasts (OFs). **(A)** The expression profile of IL-11Rα in OFs was demonstrated by immunofluorescence staining. Cells were photographed under a confocal microscope (200×). Scale bar, 50 μm. **(B)** Visualization of eight major classes of cells in orbital connective tissues by single-cell sequencing. The integrated single-cell transcriptome profiling of one patient with thyroid-associated ophthalmopathy (TAO) and one control were visualized using a t-distributed stochastic neighbor embedding (t-SNE) plot. Each point represents one cell. **(C)** Comparation of differential *IL-11Rα* gene expression in single cells (P adjust = 4.13×10^-71^). **(D)** The concentration of IL-11 in the OFs’ supernatant after a 48-h incubation without stimulus were detected by enzyme-linked immunosorbent assay (TAO, n = 8; control, n = 8). The data are expressed as the mean ± standard deviation (SD). *P < 0.05. assessed by two-tailed student’s t-test.

To examine whether IL-11 plays a role in OF biology, we investigated the expression profile of IL-11Rα in OFs. Immunofluorescence staining of OFs revealed strong expression of IL-11Rα at the protein level (**Figure 3A**). To explore the IL-11 Rα expression profile further *in vivo*, single-cell RNA sequencing of orbital connective tissues from a patient with TAO and a healthy control was performed. IL-11 Rα was highly expressed in fibroblast subsets (**Figures 3B, C**). The expression pattern of IL-11Rα indicated that fibroblasts are the predominant responders to IL-11 in the orbital connective tissue in TAO.

To investigate IL-11 expression, ELISA was performed on the culture supernatants of OFs from patients with TAO or from healthy controls. We found that IL-11 expression levels of TAO cells were greater than those control fibroblasts (P = 0.022) (**Figure 3D**). Taken together, these data provided evidence that OFs are both a source and a target of IL-11, indicating that IL-11 protein is perhaps secreted in an autocrine loop.

### IL-11 Was Secreted by OFs in Response to TGF-β1 or IL-1β

**Figure 4:**
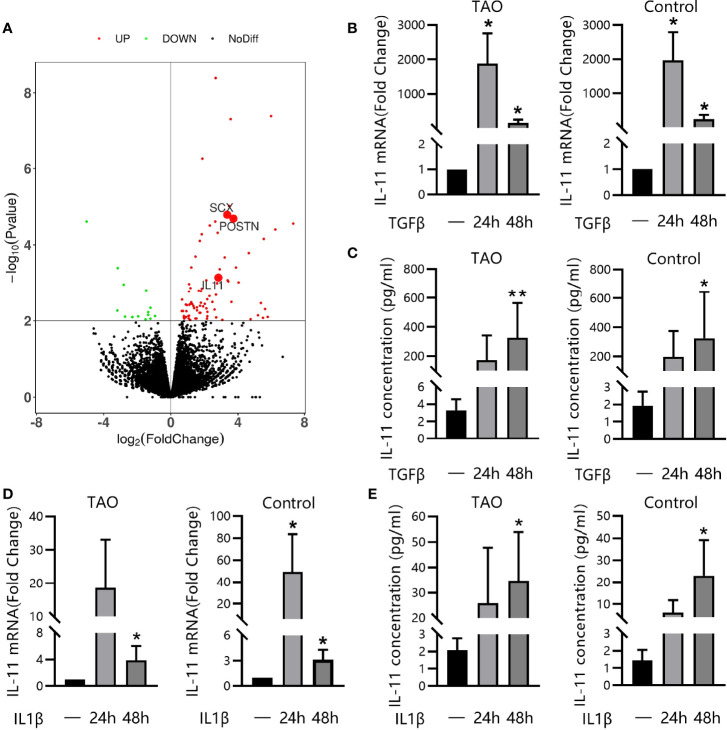
IL-11 was Secreted by OFs in Response to TGF-β1 or IL-1β. **(A)** RNA sequencing of thyroid-associated ophthalmopathy (TAO) orbital fibroblasts (OFs, n = 3) with or without TGF-β1 treatment (10 ng/mL, 48 h). DEseq2^10^-fold change in expression and P values are shown. **(B, C)** Quantitative real-time polymerase chain reaction (qRT-PCR, TAO, n = 5; control, n = 5) and enzyme-linked immunosorbent assay (ELISA, TAO, n = 8; control, n = 8) of IL-11 expression in orbital fibroblasts (OFs) after stimulation with TGF-β1(10 ng/mL, 48 h). **(D, E)** QRT-PCR (TAO, n = 6; control, n = 6) and ELISA (TAO, n = 5; control, n = 5) of IL-11 expression in orbital fibroblasts (OFs) after stimulation with IL-1β (5 ng/ml, 48 h). The data are expressed as the mean ± standard deviation (SD). *P < 0.05, **P < 0.01 as compared with the control; ns denotes no statistical significance versus the control; assessed by Tukey’s corrected one-way analysis of variance.

Transforming growth factor-beta1 (TGF-β1) represents the most prominent profibrotic cytokine and plays a pivotal role in TAO (32). To elucidate the involvement of IL-11 signaling in the profibrotic TGF-β1 signaling, RNA sequencing (RNA-seq) was performed on paired unstimulated and TGF-β1-stimulated samples. Genes were ranked based on the significance of the differences in expression levels. Among the most-upregulated genes, we detected several typical genes involved in the fibrotic process such as *SCX* (33) and *POSTN* (34). Notably, the upregulation of IL-11 expression (2.83-fold, P=7.2×10^-4^) defined the dominant transcriptional response of OFs to TGF-β1 (**Figure 4A**), which was further verified at the mRNA level by qRT-PCR and at protein levels by ELISA, both in TAO patients and in healthy controls in a time-dependent manner (**Figures 4B, C**). In addition, we stimulated OFs with other profibrotic cytokines. IL-1β significantly augmented *IL-11* mRNA and protein expression in both TAO and control subjects (**Figures 4D, E**). These results suggest that the activation of IL-11 signaling may be a downstream pathway for multiple profibrotic stimuli.

### IL-11 Induces Trans-Differentiation of OFs to Myofibroblasts

**Figure 5:**
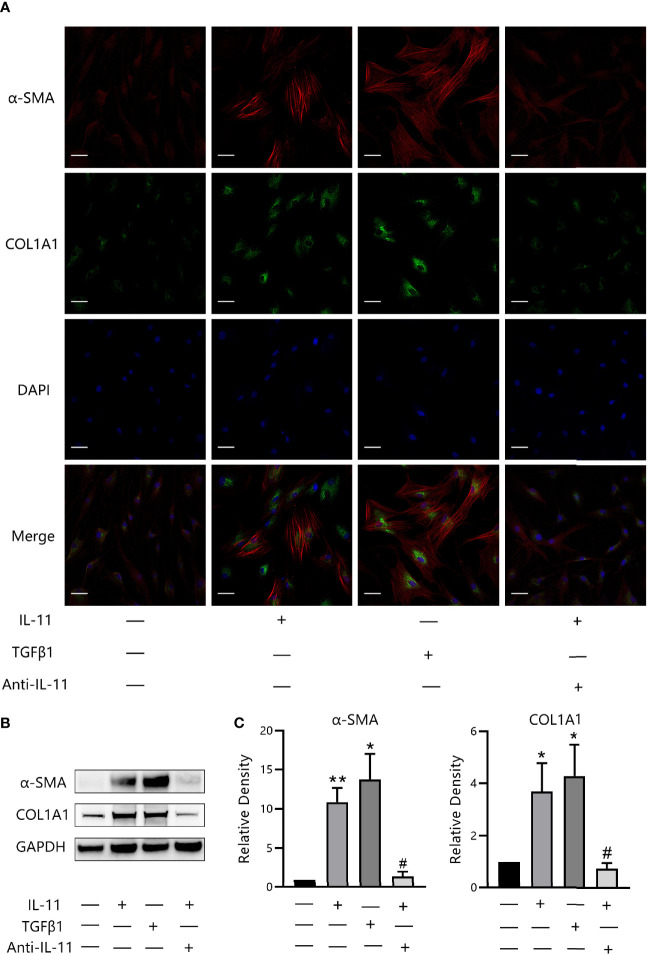
IL-11 induces trans-differentiation of orbital fibroblasts (OFs) to myofibroblasts. **(A)** Representative images of α-SMA or COL1A1 immunostaining in OFs treated with IL-11 (10 ng/mL, 48 h) or TGFβ1 (10 ng/mL, 48 h). Cells were counterstained with DAPI to visualize nuclei. Scale bars, 50 μm. **(B)** Western blot of α-SMA or COL1A1 expression in IL-11 (10 ng/mL, 48 h) or TGF-β1(10 ng/mL, 48 h, n = 4) stimulated OFs with or without a 2-h pretreatment with anti-IL-11 neutralizing antibody (anti-IL-11, 15 μg/mL). GAPDH was used as a loading control. **(C)** The protein levels were quantified and analyzed. anti-IL-11, anti-IL-11 neutralizing antibody. Data are expressed as mean ± standard deviation (SD). *P < 0.05, **P < 0.01compared with the control; ^#^P < 0.05, compared with IL-11 alone; assessed by Tukey’s corrected one-way analysis of variance.

To explore whether IL-11 is related to the profibrotic phenotype switch, we incubated OFs with IL-11 or TGF-β1. Negligible differences in mRNA levels of fibrosis-related markers (ACTA2, COL1A1, COL1A2 and FN1) were observed in IL-11 stimulated OFs (**Supplementary Figure S1**). Immunofluorescence staining revealed that IL-11 effectively boosted the formation of SMA-positive fibers and ECM protein COL1A1 (35) (**Figure 5A**), which are phenotypic hallmarks of myofibroblasts (36). In addition, we stimulated OFs with IL-11 or TGF-β1 in the presence of either an anti-IL-11 neutralizing antibody or IgG. Corroborating the immunofluorescence study, western blot analysis confirmed that IL-11 significantly induced α-SMA and COL1A1 expression, which can be abrogated by addition of anti-IL-11 neutralizing antibody (**Figures 5B, C**). These results indicated that IL-11 elicits a profibrotic response in OFs.

### IL-11 Enhances Fibrogenesis Through ERK and STAT3 Mediators in TAO

**Figure 6:**
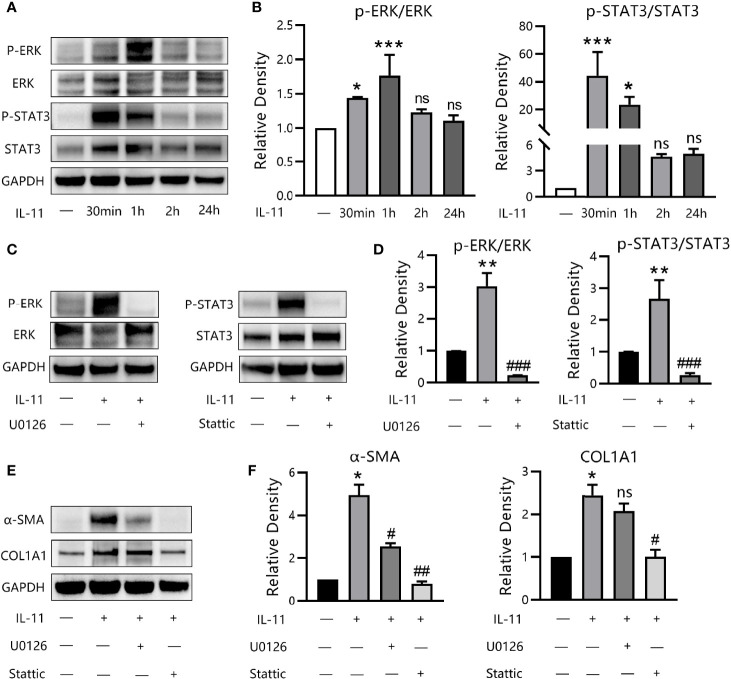
IL-11 drives fibrogenic protein synthesis *via* ERK and STAT3 pathways. **(A)** Western blots assessing protein levels of p-ERK, ERK, p-STAT3, STAT3, GAPDH in thyroid-associated ophthalmopathy (TAO) orbital fibroblasts (OFs) with the stimulation of IL-11 (10 ng/mL) for 30 min, 60 min, 2 h or 24 h (n = 3). **(B)** The protein levels were quantified and analyzed. The data are expressed as the mean ± standard deviation (SD). ***P < 0.001, *P < 0.05 compared with the control; ns denotes no statistical significance versus the control; assessed by Tukey’s corrected one-way analysis of variance. **(C)** Western blots of p-ERK, ERK, p-STAT3, STAT3, GAPDH in TAO OFs following stimulation with IL-11 (10 ng/mL, 1 h) with or without a 2-h pretreatment with U0126 (10 μM) or Stattic (10 μM) (n = 3). **(D)** The protein levels were quantified and analyzed. **(E)** Western blot of α-SMA, COL1A1, and GAPDH expression in TAO OFs under IL-11 (10 ng/mL, 48 h) stimulation with or without a 2-h pretreatment with U0126 (10 μM) or Stattic (10 μM) (n = 4). **(F)** The protein levels were quantified and analyzed. Data are expressed as mean ± SD. **P < 0.01, *P < 0.05 compared with the control; ^###^P < 0.001, ^##^P < 0.01, ^#^P < 0.05 compared with IL-11 alone; ns denotes no statistical significance versus IL-11; assessed by Tukey’s corrected one-way analysis of variance.

To decipher the signaling mechanisms underlying the profibrotic effects of IL-11 in OFs, several classical pathways were screened. We first detected the phosphorylation level of proteins in the canonical (STAT3) and noncanonical (ERK, SMAD2/3, p38/MAPK, JNK/MAPK) signaling pathways for different durations, by western blotting. Exposure of OFs to IL-11 resulted in the phosphorylation of ERK and STAT3 (**Figures 6A, B**), whereas the STAT3 pathway appeared maximally activated at earlier time points, with a peak at 30 min. However, SMAD2/3, p38/MAPK, and JNK/MAPK were not phosphorylated after IL-11 stimulation (data not shown). In addition, the increase in phosphorylation of ERK and STAT3 by IL-11 could be suppressed by pharmacological inhibition of ERK (U0126) or STAT3 (Stattic) (**Figures 6C, D**).

To dissect this in further detail, we investigated the effects of U0126 and Stattic on IL-11-induced α-SMA and COL1A1 expression. Western blot results revealed that inhibition of STAT3 was equally effective in suppressing α-SMA expression *via* ERK inhibition. Additionally, COL1A1 expression was abrogated by Stattic and was partly attenuated by U0126, although this was not significant (**Figures 6E, F**). Thus, our data demonstrated that IL-11 induces OF phenotypic switching *via* activation of the ERK and STAT3 pathways.

## Discussion

To the best of our knowledge, the expression pattern and exact biological function of IL-11 in TAO have not been described to date. In this study, we showed that IL-11 levels were higher in the serum and orbital connective tissues in patients with TAO than in those of healthy controls, and that these levels correlated positively with disease activity. Single-cell RNA sequencing of orbital connective tissue indicated that IL-11Rα was predominantly expressed in OFs. RNA sequencing of paired unstimulated and TGF-β1-stimulated samples revealed that upregulation of IL-11 expression defined the dominant transcriptional response. We also demonstrated that IL-11 signaling occurred downstream of TGF-β1 and IL-1β. Our results indicated that IL-11 protein is secreted in an autocrine loop in TAO. Moreover, we showed that IL-11 mediated the profibrotic phenotype switch, based on the expression of myofibroblast differentiation markers, which could be abrogated by an anti-IL-11-neutralizing antibody. Furthermore, we showed that ERK, but not STAT3, plays an important role in the pro-fibrotic, translationally specific signaling activity of IL-11.

Previous studies have indicated that IL-11 exerts a protective, anti-inflammatory, and antifibrotic role, based on observations in a variety of organs in mouse models (37–40). In contrast, Tang et al. indicated that IL-11 was overexpressed in Club/Clara cells and consequently induced airway inflammation and subepithelial fibrosis (41). Chen et al. suggested that pathological lung remodeling induced by IL-13 is dependent on the upregulation of endogenous IL-11 expression (42). Moreover, Stuart et al. utilized the species-matched IL-11 and verified the profibrotic function of IL-11 in the liver, kidney, dermis, and cardiovascular system (23, 25, 28, 43). Their interpretation of the confounding IL-11 function is that extrinsic rhIL-11 provides a negative feedback mechanism for endogenous murine IL-11 in the context of IL-11-driven diseases in mice. Thus, endogenous mouse IL-11 was downregulated, which contributed to the observed therapeutic effect. Instead, administration of species-matched recombinant mouse IL-11 resulted in the activation of mouse cardiac and renal fibroblasts (44).

Consistent with previous studies on other autoimmune diseases, such as multiple sclerosis (28), rheumatoid arthritis (45), and severe asthma (46), our data demonstrated that circulating IL-11 was overexpressed in TAO patients than in healthy controls and was correlated positively with disease activity. Therefore, serum IL-11 levels may serve as a potential biomarker for the severity of TAO. Additionally, we identified that IL-11 expression was elevated in the local orbital connective tissues of patients with TAO and correlated positively with the clinical progression of TAO. These systemic and local associations between IL-11 levels and CAS suggested that IL-11-mediates pathogenic changes in TAO.

Although IL-11 belongs to the IL-6 family of cytokines, several studies have revealed that IL-11Rα differs from IL-6R in its expression pattern across cell types, and that IL-11 and IL-6 can activate distinct target cell types (23). Unlike IL-6R, which is predominantly expressed on the surface of immune cells, IL-11Rα is primarily expressed on stromal cells comprising fibroblasts, adipocytes, epithelial cells, and vascular smooth muscle cells (23, 25, 43, 47). Corroborating these findings, single-cell sequencing results in the present study revealed that the OFs most strongly expressed IL-11Rα.

It has been demonstrated that stromal fibroblasts are the major source of IL-11 in the lung, heart, and gastrointestinal tract under the stimulation of other cytokines, such as TGF-β, IL-1β, and IL-22 (48). Consistent with these findings, we determined, by RNA sequencing, that IL-11 is one of the dominant transcriptional responses to TGF-β1. Moreover, we observed that TGF-β1 or IL-1β could induce the expression of IL-11 in OFs over time. These results are in accordance with the previous concept that IL-11 is a necessary downstream factor for multiple profibrotic stimuli (25).

TGF-β1 has been reported to be the most potent inducer of myofibroblast differentiation, which is characterized by increased α-SMA expression (49). Several studies have shown that IL-11 is a regulatory target of TGF-β1: upregulation of IL-11 contributes to the activation of myofibroblasts, which is a crucial profibrotic pathway in cardiac fibrosis, pulmonary fibrosis, and liver fibrosis (23, 25, 43). Hence, we considered that IL-11 may also play a pro-fibrotic role in TAO. In the current study, we demonstrated that stimulating OFs with rhIL-11 strongly promoted fibrosis by increasing myofibroblast numbers and ECM production. In addition, we explored whether IL-11 is the dominant factor required for the pro-fibrotic effect of TGF-β1. We pretreated OFs with anti-IL-11 neutralizing antibody with subsequent administration of TGF-β1. However, our data differed from those reported for cardiac fibrosis (23) and pulmonary fibrosis (25). The expression of α-SMA or COL1A1 was not reduced by IL-11 neutralization (data not shown). We hypothesized that this might be due to the cell-to-cell difference. Further investigation is needed to determine the mechanistic interaction between TGF-β1 and IL-11.

It has been reported that the downstream pathway of IL-11 may vary across different tissue contexts (50). In this study, we demonstrated that IL-11 stimulates ERK and STAT signaling. However, some unexpected results were revealed by our study, as both IL-6 and IL-11 primarily exert their biological effects by interacting with a similar gp130 homodimer, which signals predominantly *via* the canonical JAK/STAT signaling pathway downstream of IL-6 to induce a pro-inflammatory response at the transcript level (44). Our data illustrated that, in OFs, IL-11 enhanced the expression of proteins with profibrogenic properties, and we observed very little effect of IL-11 at mRNA levels. Corden et al. proposed that IL-11 stimulation promoted the expression of pro-fibrotic genes at the post-transcriptional level. This effect relies on the phosphorylation of ERK, and subsequently regulates its downstream substrates, such as 40S ribosomal protein S6 kinase and eukaryotic translation initiation factor 4E, which participate in the activation of protein translation (24). Other studies have also implicated that IL-11 mediation of the pro-fibrotic process depends on activation of the non-canonical ERK signaling pathway. In contrast, STAT, which is considered the canonical signaling pathway, is only transiently and mildly phosphorylated. Consequently, the effect of IL-11 was undetectable at the transcriptional level. Consistent with these studies, we also observed sustained ERK activation after IL-11 administration, with transient phosphorylation of STAT3 in 2 hours.

In addition, we determined that pharmacological inhibition of STAT3 was equally effective in blocking the phenotypically fibrotic transition as ERK inhibition in OFs. Considering our previous findings, this was unexpected, but was in line with the literature (51, 52). STAT3 activity has been implicated in proteotoxicity due to overloading of the endoplasmic reticulum. It has been demonstrated that Stattic prevented IL-11-induced fibrogenesis by inhibiting both ERK and STAT3 (53). The extent to which STAT3 and ERK interact under IL-11 stimulation in OFs is currently being investigated. However, collectively, the mechanism described above provides evidence that ERK may be a crucial factor in the pro-fibrotic, translationally specific signaling activity of IL-11, while STAT3 appears to be non-essential.

A growing body of evidence indicates that blockage of IL-11 signaling is not associated with significant side effects, as the expression level of IL-11 in healthy tissue is almost undetectable (54). In agreement with this, humans or mice with loss-of-function mutations in IL-11 or IL-11Rα do not suffer from serious disorders. Only those with biallelic IL-11Rα mutations sometimes manifest abnormalities, such as mild craniosynostosis, scoliosis, joint laxity, and delayed tooth eruption, but are still otherwise healthy (55–57). As IL-11 has limited effects on healthy individuals and organs, we deduce that inhibition of IL-11 may also have few adverse effects in patients with TAO.

The current study had some limitations. First, to elucidate the definite differences in IL-11 expression in serum between individuals with TAO or GD, we plan to enroll a patient cohort with GD but without TAO in a further study with a larger sample size. Second, further investigation is necessary to determine the ERK-dependent downstream molecular mechanisms of IL-11 signaling in TAO. Third, in this study, the profibrotic effects of IL-11 in TAO have only been studied *in vitro*. To elucidate the biological functions of IL-11 *in vivo*, further experimental exploration based on the TAO animal models is needed (58).

## Conclusion

In conclusion, our data illustrated that IL-11 is involved in the pathological process of TAO and its levels correlate positively with disease activity, implying that it may be used as a potential biomarker for the severity of TAO. Additionally, we demonstrated IL-11Rα and IL-11 were co-expressed in OFs, and multiple profibrotic stimuli could prompt the expression of IL-11. Furthermore, our data showed that IL-11 induced the profibrotic phenotypic transition of OFs, predominantly mediated by activation of ERK in a post-transcriptional manner. The results of this study suggest the potential of IL-11 as a therapeutic target candidate for the treatment of TAO.

## Data Availability Statement

The datasets presented in this study can be found in online repositories. The name of the repository and accession number can be found below: https://www.ncbi.nlm.nih.gov/geo/, accession ID: GSE194324.

## Ethics Statement

The studies involving human participants were reviewed and approved by Ethics Committee of the Zhongshan Ophthalmic Center, Sun Yat-sen University. The patients/participants provided their written informed consent to participate in this study.

## Author Contributions

PW and BL are jointly responsible for experiment design, experiment implementation, and manuscript writing. SH, JM, FZ, and MZ are responsible for experiment implementation and critical data discussion. YK and XH are responsible for sample collecting and literature searching. HY and DH are responsible for the overall control of the experiment and revision of the manuscript. All authors contributed to the article and approved the submitted version.

## Funding

This study was supported by Guangdong Provincial Natural Science Foundation (2021A1515010467) and Five-year Plan Project of Zhongshan Ophthalmic Center (3030901010071).

## Conflict of Interest

The authors declare that the research was conducted in the absence of any commercial or financial relationships that could be construed as a potential conflict of interest.

## Publisher’s Note

All claims expressed in this article are solely those of the authors and do not necessarily represent those of their affiliated organizations, or those of the publisher, the editors and the reviewers. Any product that may be evaluated in this article, or claim that may be made by its manufacturer, is not guaranteed or endorsed by the publisher.
