# Supplementary material for: IL-11 Is Elevated and Drives the Profibrotic Phenotype Transition of Orbital Fibroblasts in Thyroid-Associated Ophthalmopathy
Source: Front Endocrinol (Lausanne). 2022 Feb 22;13:846106. doi: 10.3389/fendo.2022.846106 (PMC8902078; doi:10.3389/fendo.2022.846106)
Supplement: Supplementary file 1 [file Image_1.pdf]

Supplementary materials:

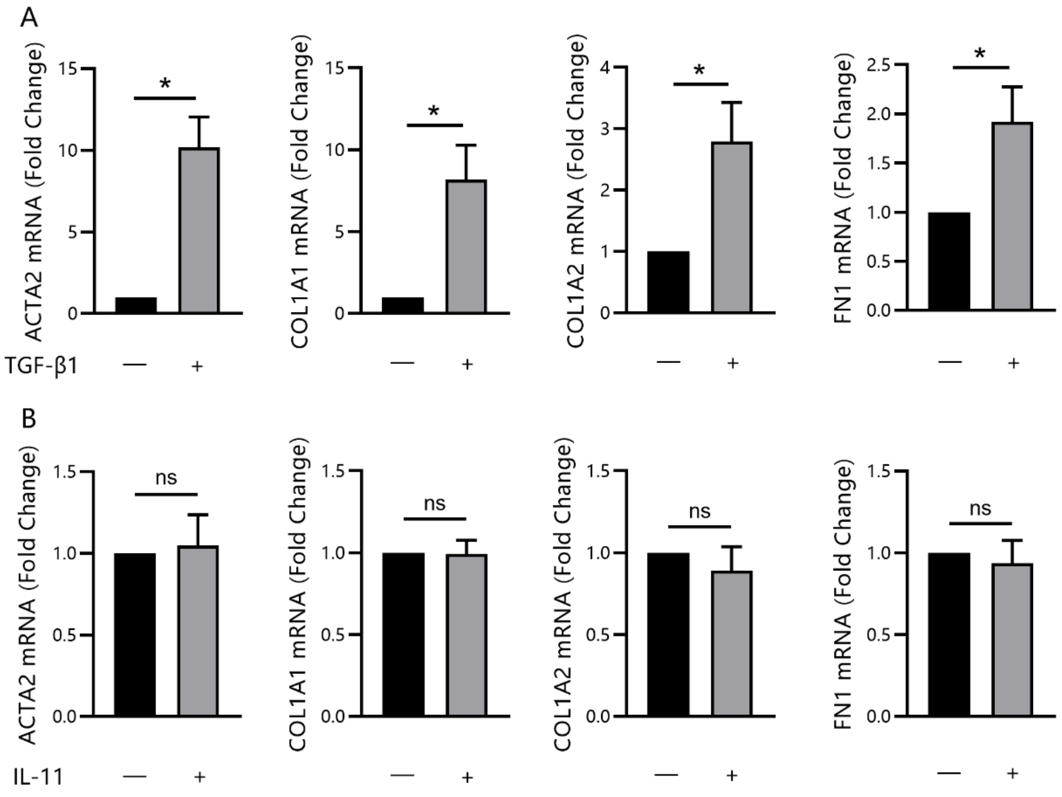

Supplementary Figure S1. No effect of IL-11 on expression of fibrosis-related markers was observed.

(A, B) Quantitative real-time polymerase chain reaction (qRT-PCR) of ACTA2, COL1A1, COL1A2 and FN1 expression in orbital fibroblasts (OFs) after stimulation with TGF-β1 (10ng/ml, 24 h) or IL-11 (10ng/ml, 24 h). TAO, n = 3; Control, n = 3. The data are expressed as the mean ± standard deviation (SD). * $P < 0.05$ as compared with the control; ns denotes no statistical significance; assessed by Mann-Whitney test.
